# Differential Effects of Sphingolipids on Cell Death and Antioxidant Defenses in Type 1 and Type 2 Endometrial Cancer Cells

**DOI:** 10.3390/ijms26104472

**Published:** 2025-05-08

**Authors:** Agnieszka U. Błachnio-Zabielska, Patrycja Sadowska, Urszula Chlabicz, Karolina Pogodzińska, Hervé Le Stunff, Piotr Laudański, Jacek Szamatowicz, Mariusz Kuźmicki

**Affiliations:** 1 Department of Hygiene, Epidemiology and Metabolic Disorders, Medical University of Bialystok, 15-089 Bialystok, Poland; 2 CNRS UMR 9197, Institut des Neurosciences Paris-Saclay, Université Paris-Saclay, 91400 Saclay, France; 3 Department of Obstetrics, Gynecology and Gynecological Oncology, Medical University of Warsaw, 02-091 Warsaw, Poland; 4 Women’s Health Research Institute, Calisia University, 62-800 Kalisz, Poland; 5 OVIklinika Infertility Center, 01-377 Warsaw, Poland; 6 Department of Gynecology and Gynecological Oncology, Medical University of Bialystok, 15-089 Bialystok, Poland

**Keywords:** endometrial cancer, ceramide, sphingosine-1-phosphate, sphingolipid rheostat, cell viability, cell death

## Abstract

Endometrial cancer (EC) is classified into two main subtypes with distinct molecular profiles. Sphingolipids, particularly ceramide and sphingosine-1-phosphate (S1P), are crucial regulators of cell survival, apoptosis, and oxidative stress. This study examined the impact of sphingolipid metabolism in Ishikawa (type 1) and HEC-1A (type 2) EC cells following the silencing of *Sptlc1* and *Sptlc2*, which encode subunits of serine palmitoyltransferase (SPT), a key enzyme in de novo sphingolipid synthesis. Gene silencing was confirmed by RT-PCR and Western blot, while sphingolipid levels were quantified using UHPLC/MS/MS and the sphingolipid rheostat (S1P/ceramide ratio) was calculated. Cell viability (MTT assay), cell death, ROS levels (ELISA), total antioxidant capacity (TAC), catalase and caspase-3 activity, and mitochondrial membrane potential were also assessed. The obtained data showed higher ceramide levels in Ishikawa_(CON)_ cells and higher S1P levels in HEC-1A_(CON)_ cells, resulting in a higher sphingolipid rheostat in HEC-1A cells. SPT knockdown reduced sphingolipid levels, increased cell viability, elevated ROS levels, and decreased cell death, particularly in Ishikawa cells. Furthermore, after gene silencing, these cells exhibited reduced catalase activity and diminished TAC, indicating an impaired redox balance. These findings reveal subtype-specific responses to disrupted sphingolipid synthesis and highlight the importance of sphingolipid homeostasis in the behavior of EC cells.

## 1. Introduction

Endometrial cancer (EC) is one of the most prevalent gynecological malignancies, with its incidence steadily increasing worldwide [1]. It is classified into two distinct subtypes based on histopathological and molecular characteristics: type 1 and type 2 [2]. Type 1 endometrial cancer, accounting for approximately 80% of cases, is estrogen-dependent, typically low-grade, and associated with favorable prognosis. It often arises in the context of endometrial hyperplasia and is frequently linked to metabolic disorders such as obesity and diabetes [3]. In contrast, type 2 endometrial cancer is estrogen-independent, high-grade, and more aggressive, with a poorer clinical outcome. This subtype includes serious and clear cell carcinomas, which exhibit a higher propensity for metastasis and resistance to conventional therapies [4]. Despite advances in understanding the molecular basis of endometrial cancer, the mechanisms underlying its progression and subtype heterogeneity remain incompletely understood, highlighting the need for further research into the metabolic and signaling pathways that drive these processes. One emerging area of interest in cancer biology is sphingolipid metabolism, which plays a crucial role in regulating key cellular processes such as proliferation, apoptosis, migration, and angiogenesis [5]. Among sphingolipids, ceramide and sphingosine-1-phosphate (S1P) play particularly significant but opposing roles in cancer progression. Ceramide, often referred to as a pro-apoptotic lipid, induces cell cycle arrest and promotes apoptosis, making it a key mediator of stress responses and chemotherapy-induced cell death [6]. Ceramide accumulation is known to trigger mitochondrial outer membrane permeabilization, leading to the loss of mitochondrial membrane potential and the activation of downstream effectors such as caspase-3 [7,8]. Caspase-3, a key executioner caspase, cleaves multiple cellular substrates, culminating in controlled cell death. Disruption of mitochondrial potential is an early and sensitive indicator of intrinsic apoptotic signaling, often modulated by changes in the sphingolipid rheostat [9]. Conversely, S1P, generated by the phosphorylation of sphingosine, exerts pro-survival and pro-proliferative effects, contributing to tumor growth, angiogenesis, and metastasis [10]. The balance between ceramide and S1P, known as the “sphingolipid rheostat”, is tightly regulated and has been implicated in the development and progression of various cancers, including endometrial cancer [11]. Dysregulation of this balance, favoring S1P over ceramide, is frequently observed in cancer cells and is associated with increased aggressiveness and therapy resistance [6]. A key regulator of sphingolipid metabolism is serine palmitoyltransferase (SPT), the first and rate-limiting enzyme in the de novo synthesis of sphingolipids. SPT catalyzes the condensation of serine and palmitoyl-CoA to form 3-ketosphinganine, thereby controlling the initial step of sphingolipid biosynthesis [12]. By controlling the initial step of sphingolipid biosynthesis, SPT plays a crucial role in determining the levels of downstream sphingolipids, including ceramide and S1P, whose balance dictates cell fate.

Inhibition or silencing of SPT has been shown to disrupt sphingolipid metabolism, leading to altered ceramide and S1P levels, which can profoundly impact cancer cell behavior [5]. However, the specific role of SPT and sphingolipid metabolism in the context of endometrial cancer subtypes remains poorly understood. In addition to sphingolipid metabolism, oxidative stress has been increasingly recognized as a key player in cancer progression. Reactive oxygen species (ROS), byproducts of cellular metabolism, influence cancer biology through dual mechanisms. At moderate levels, ROS promote cell proliferation and survival by activating pro-tumorigenic signaling pathways [13]. However, excessive ROS accumulation can lead to oxidative damage to DNA, proteins, and lipids, ultimately inducing cell death [14]. Enzymatic antioxidants such as catalase play a crucial role in neutralizing ROS and maintaining redox homeostasis [15]. Reduced catalase activity has been associated with increased oxidative underscoring its role in cellular adaptation to redox imbalance [16].

The interplay between sphingolipid metabolism and oxidative stress is particularly intriguing, as S1P has been shown to mitigate oxidative stress through its pro-survival effects [5]. This complex relationship suggests that targeting sphingolipid metabolism could modulate oxidative stress levels, thereby influencing cancer cell behavior. Given these insights, the aim of this study was to explore the role of sphingolipid metabolism in both types of endometrial cancer (type 1 and type 2). We sought to enhance our understanding of the molecular mechanisms underlying endometrial cancer heterogeneity and progression.

## 2. Results

### 2.1. Gene Silencing

**Figure 1:**
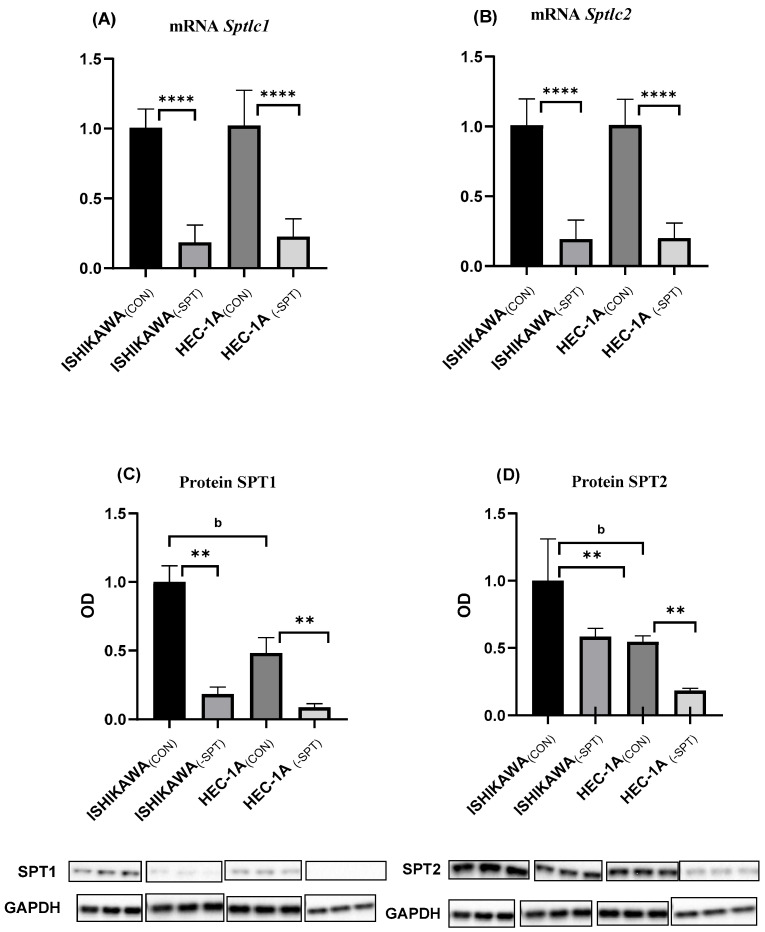
(**A**) The impact of *Sptlc1* gene silencing on *Sptlc1* mRNA in Ishikawa and HEC-1A cells; (**B**) The impact of *Sptlc2* gene silencing on *Sptlc2* mRNA in Ishikawa and HEC-1A cells; (**C**) The impact of *Sptlc1* gene silencing on SPT1 protein content in Ishikawa and HEC-1A cells; (**D**) The impact of *Sptlc2* gene silencing on SPT2 protein content in Ishikawa and HEC-1A cells; Data are presented as medians (interquartile range). The difference between groups and compared by Mann–Whitney U test: ** *p* < 0.01, **** *p* < 0.0001 vs. control group; ^b^ *p* < 0.01, vs. Ishikawa_(CON)_ group.

RT-PCR assay confirmed a significant reduction of Sptlc1 and Sptlc2 mRNA expression in both Ishikawa and HEC-1A silenced cells as compared to non-silenced control cells (*p* < 0.0001 in all cases) (Figure 1A). Silencing the Sptlc1 and Sptlc2 genes in Ishikawa and HEC-1A cell lines also reduced the content of SPT1 (*p* < 0.01 for both cell lines) and SPT2 proteins (*p* < 0.001, for both cell lines) (Figure 1B).

### 2.2. Sphingolipids Content and Sphingolipid Rheostat

Total ceramide levels were significantly higher in Ishikawa_(CON)_ cells compared to HEC-1A_(CON)_ cells (*p* < 0.0001). When examining individual ceramide species, the levels of C18:1-Cer, C18:0-Cer, C24:1-Cer, and C24:0-Cer were significantly elevated in Ishikawa_(CON)_ cells compared to HEC-1A_(CON)_ cells. In contrast, the level of C16:0-Cer was significantly lower in Ishikawa_(CON)_ cells compared to HEC-1A_(CON)_ cells, while the levels of C14:0-Cer, C20:0-Cer, and C22:0-Cer did not differ significantly between the two cell lines. Additionally, the levels of Sph and SPA were significantly higher in Ishikawa_(CON)_ cells than in HEC-1A_(CON)_ cells. Notably, the level of S1P was significantly higher in HEC-1A_(CON)_ cells compared to Ishikawa_(CON)_ cells (Table 1).

Silencing the genes encoding the two subunits of SPT (*Sptlc1* and *Sptlc2*) resulted in a reduction in total ceramide levels in both cell types. In Ishikawa cells, silencing led to a decrease in the levels of all measured sphingolipids except for C14:0-Cer, C20:0-Cer, and C22:0-Cer, which remained unchanged compared to non-silenced cells. Similarly, in HEC-1A cells, *Sptlc1* and *Sptlc2* genes’ silencing caused a reduction in the levels of all measured sphingolipids except for C14:0-Cer, which showed no significant change compared to non-silenced cells. In HEC-1A cells, representing type 2 endometrial cancer, the S1P level decreased slightly but significantly after silencing the genes encoding the two SPT subunits compared to non-silenced cells (Table 1). Additionally, the sphingolipid rheostat value was higher in HEC-1A_(CON)_ cells for both the total ceramide level and individual ceramides (except S1P/C16:0-Cer, which was lower) compared to the corresponding values in Ishikawa_(CON)_ cells. In Ishikawa cells, silencing the genes encoding the two SPT subunits did not significantly affect the sphingolipid rheostat value for the total ceramide and C24:1-Cer levels. However, it decreased this index for C14:0-Cer, C20:0-Cer, and C22:0-Cer, while increasing it for C16:0-Cer, C18:1-Cer, C18:0-Cer, and C24:0-Cer. In HEC-1A cells following SPT silencing, the rheostat value for C14:0-Cer, C18:1-Cer, and C24:0-Cer remained unchanged, while an increase in this index was observed for C16:0-Cer, C18:0-Cer, and C24:1-Cer (Table 2).

### 2.3. Cell Viability and Death

**Figure 2:**
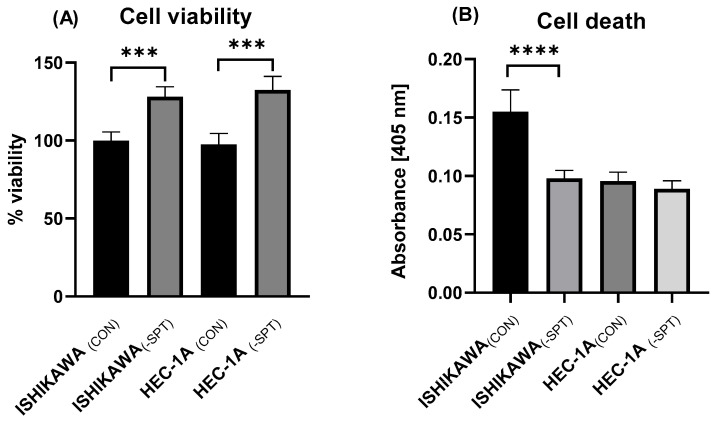
(**A**) The impact of *Sptlc1* and *Sptlc2* gene silencing on cell viability in Ishikawa and HEC-1A cells; (**B**) The impact of *Sptlc1* and *Sptlc2* gene silencing on cell death in Ishikawa and HEC-1A cells. Data are presented as medians (interquartile range). The difference between groups and compared by Mann–Whitney U test: *** *p* < 0.001, **** *p* < 0.0001 vs. control group.

The knockdown of genes encoding two subunits of SPT resulted in a notable increase in cell viability in both cell lines. Specifically, cell viability increased by 28% in Ishikawa cells (*p* < 0.001) and by 33% in HEC-1A cells (*p* < 0.001) compared to non-silenced control (Figure 2A). Furthermore, silencing the genes encoding the SPT subunits significantly reduced cell death in the Ishikawa cell line (*p* < 0.0001). In contrast, this intervention did not significantly affect cell death in HEC-1A cells. These results suggest that the suppression of SPT subunit expression differentially affects cell death in the two endometrial cancer cell lines, with a more pronounced impact on Ishikawa cells (Figure 2B).

### 2.4. Correlation Between the Shingolipid Rheostate and Cell Death

In Ishikawa cells, moderate and strong positive correlations were observed between cell death and sphingolipid rheostat values for S1P/C14:0-Cer (r = 0.61; *p* < 0.01), S1P/C16:0-Cer (r = 0.47; *p* < 0.05), S1P/C20:0-Cer (r = 0.50; *p* < 0.05), and S1P/C22:0-Cer (r = 0.57; *p* < 0.05). In contrast, S1P/C18:1-Cer (r = –0.71; *p* < 0.01), S1P/C18:0-Cer (r = –0.61; *p* < 0.01), and S1P/C24:0-Cer (r = –0.86; *p* < 0.0001) were inversely correlated with cell death, suggesting subtype- or chain length-specific effects of ceramide species on apoptosis. Interestingly, the correlation between total ceramide rheostat (S1P/Total Cer) and cell death was negligible (r = 0.002), indicating that individual ceramide species may be more informative than total ceramide levels in predicting functional outcomes. In HEC-1A cells, most correlations were weak and the only statistically significant correlation was found for S1P/C22:0-Cer (r = 0.50; *p* < 0.05). No clear trend was observed for the total rheostat (r = –0.16) (Table 3).

### 2.5. Reactive Oxygen Species (ROS) Levels, Catalase Activity, and Total Antioxidant Capacity (TAC)

**Figure 3:**
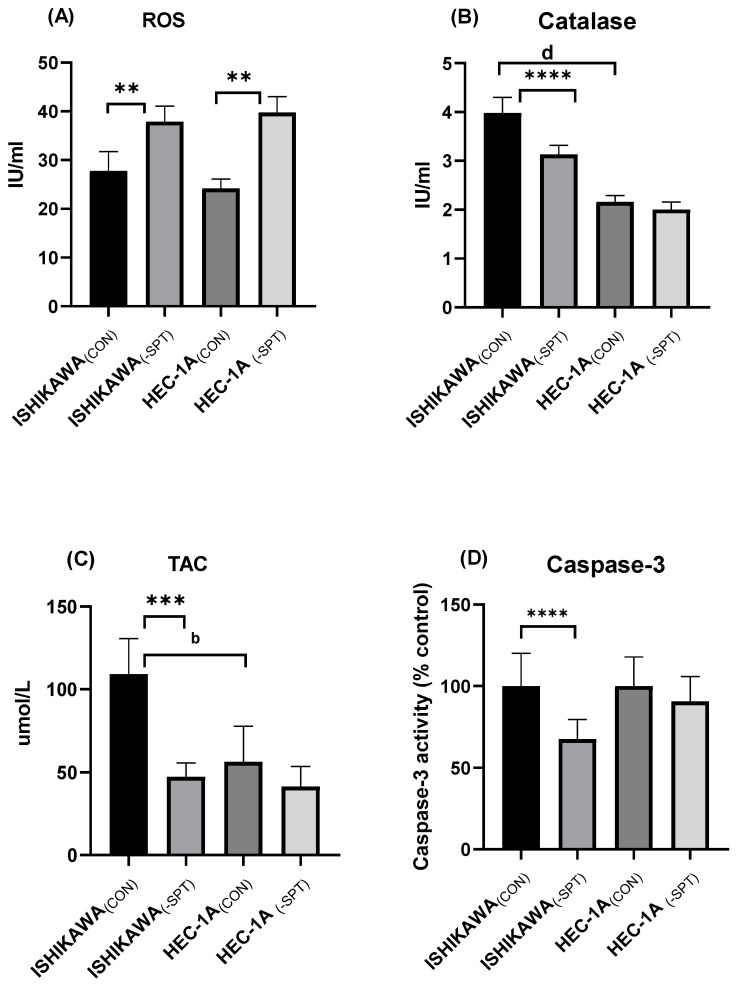
(**A**) The impact of *Sptlc1* and *Sptlc2* gene silencing on ROS levels in Ishikawa and HEC-1A cells; (**B**) The impact of *Sptlc1* and *Sptlc2* gene silencing on catalase activity in Ishikawa and HEC-1A cells; (**C**) The impact of *Sptlc1* and *Sptlc2* gene silencing on TAC in Ishikawa and HEC-1A cells; (**D**) The impact of *Sptlc1* and *Sptlc2* gene silencing on caspase-3 activity in Ishikawa and HEC-1A cells; Data are presented as medians (interquartile range). The difference between groups and compared by Mann–Whitney U test: ** *p* < 0.01, *** *p* <0.001, **** *p* < 0.0001 vs. control group; ^b^ *p* < 0.01, ^d^ *p* < 0.0001 vs. Ishikawa_(CON)_ group.

No significant differences in ROS levels were observed between Ishikawa_(CON)_ and HEC-1A_(CON)_ cell lines, although slightly higher concentrations were detected in Ishikawa cells. Silencing of genes encoding SPT1 and SPT2 subunits resulted in a significant increase in ROS levels in both cell lines, though the overall levels remained within a low range (Figure 3A). Furthermore, in Ishikawa_(CON)_ cells, catalase activity was significantly higher compared to HEC-1A_(CON)_ cells (3.84 U/mL vs. 2.21 U/mL, *p* < 0.0001). Following SPT subunit gene silencing, the activity of this enzyme decreased by approximately 18.6% (*p* < 0.0001) in Ishikawa cells and by about 11% in HEC-1A cells (Figure 3B). Additionally, Ishikawa_(CON)_ cells exhibited a markedly higher TAC compared to HEC-1A_(CON)_ cells. However, silencing of the SPT subunit genes resulted in a significant reduction of TAC in Ishikawa cells (Figure 3C).

### 2.6. Caspase-3 Activity and Mitochondrial Potential

In Ishikawa cells, silencing of the genes encoding the two SPT subunits resulted in a significant reduction of caspase-3 activity by over 32% (*p* < 0.0001). In contrast, in HEC-1A cells, only a slight reduction in the activity of this enzyme was observed (less than 10%), which did not reach statistical significance (Figure 3D). Furthermore, in Ishikawa cells, silencing of the genes encoding SPT subunits reduced the total population of cells with mitochondrial depolarization by 31, 44% (*p* < 0.0001) compared to non-silenced cells, indicating improved mitochondrial integrity following SPT suppression. In HEC-1A cells, SPT silencing also led to a decrease in the percentage of cells with mitochondrial depolarization, although the reduction was significantly smaller than that observed in Ishikawa cells (Table 4).

## 3. Discussion

Endometrial cancer is the most common gynecological malignancy, and its pathogenesis is influenced by various molecular factors, including lipid metabolism [17]. In this study, we investigated the role of sphingolipids in two endometrial cancer cell lines, Ishikawa and HEC-1A, which represent type 1 and type 2 endometrial cancer, respectively. By silencing the *Sptlc1* and *Sptlc2* genes in both cell lines, we aimed to disrupt de novo sphingolipid synthesis and examine the resulting changes in sphingolipid profiles, as well as in key cellular processes, including cell viability, cell death, ROS levels, catalase activity, total antioxidant capacity, caspase-3 activity, and mitochondrial potential. This study revealed distinct sphingolipid profiles in two representative endometrial cancer cell lines. Our previous research demonstrated that ceramide levels in type 1 endometrial cancer tissue were significantly higher compared to those in healthy endometrial tissue [18]. In the present study, Ishikawa cells exhibited significantly higher ceramide levels than HEC-1A cells, further supporting these earlier findings. Numerous studies have identified altered sphingolipid metabolism as a hallmark of various cancers, including endometrial cancer [19,20,21,22]. Published data also indicate that ceramide levels vary significantly depending on the type of cancer. For instance, reduced ceramide concentrations have been reported in aggressive glioblastoma multiforme [23,24,25,26], human astrocytoma [26], ovarian carcinoma [27], and colon cancer [28], whereas elevated ceramide levels have been observed in breast cancer [29,30], pancreatic cancer [31], and human head and neck squamous cell carcinoma [32]. Notably, studies on breast cancer [30] have demonstrated higher ceramide levels in estrogen receptor-positive breast cancer compared to estrogen receptor-negative cases, suggesting that increased sphingolipid concentrations may correlate with lower tumor aggressiveness. These findings are consistent with our observations, as Ishikawa cells exhibited higher ceramide levels than HEC-1A cells. However, emerging evidence indicates that not all ceramides exert identical cellular functions. The biological roles of ceramide in cancer progression vary substantially depending on its acyl chain length and saturation. It has been found that C18:0-Cer promotes apoptosis, whereas C16:0-Cer displays anti-apoptotic properties [33,34,35,36]. Our results align with these findings, as Ishikawa cells, which represent less aggressive type 1 endometrial cancer, exhibited higher levels of C18:0-ceramide and lower levels of C16:0-ceramide compared to the more aggressive HEC-1A cells. Beyond chain length, the degree of unsaturation, particularly the presence of double bonds in the acyl chain, also plays an important role in determining ceramide function. Unsaturated ceramides exhibit distinct biophysical and cellular properties. The double bonds disrupt the tight packing of lipid bilayers and affect membrane organization, particularly the formation of lipid rafts and ceramide-rich microdomains, thereby modulating downstream signaling pathways including apoptosis, autophagy, and inflammatory responses [37,38,39,40,41,42,43,44]. In our study, both saturated and unsaturated ceramides were elevated in Ishikawa cells, suggesting a global upregulation of ceramide biosynthesis in these cells. In contrast to ceramide, the level of S1P, a pro-proliferative lipid, was significantly higher in HEC-1A cells than in Ishikawa cells. This observation aligns with the more aggressive nature of type 2 endometrial cancer and underscores the role of S1P in enhancing survival, proliferation, and metastatic potential [10,45]. The observed differences in ceramide and S1P content between the two cell types influenced the sphingolipid rheostat, which is defined as the balance between S1P and ceramide. Notably, the sphingolipid rheostat was significantly higher in HEC-1A cells than in Ishikawa cells, favoring cell proliferation and survival in HEC-1A cells, whereas the lower rheostat in Ishikawa cells promoted apoptosis [5]. The literature data suggest that tumor aggressiveness may be determined more by the sphingolipid rheostat than by absolute ceramide levels. For instance, gliomas with isocitrate dehydrogenase (IDH) mutations, which are associated with lower malignancy [46], exhibit reduced sphingosine kinase 1 activity. This leads to decreased S1P levels, shifting the sphingolipid rheostat toward lower values and thereby promoting apoptosis in this glioma subtype [23].

Similarly, reactive oxygen species in cancer cells can exert opposing functions depending on their concentration. At low to moderate levels, ROS promote tumor proliferation, whereas at high concentrations, they induce cell death [13,14,47,48]. Interestingly, in our study, no significant differences in ROS levels were observed between the two cell types. Similar findings were reported in another study, which also found no differences in ROS levels between Ishikawa and HEC-1A cells [49]. However, we observed a higher total antioxidant capacity in Ishikawa_(CON)_ cells compared to HEC-1A_(CON)_ cells, which likely reflects their greater inherent ability to counteract oxidative stress. Consistent with the TAC results, catalase activity was significantly higher in Ishikawa_(CON)_ cells compared to HEC-1A_(CON)_ cells. These findings suggest that Ishikawa cells possess a more robust enzymatic antioxidant system, which may contribute to their enhanced ability to maintain redox homeostasis.

Silencing of the *Sptlc1* and *Sptlc2* genes resulted in a significant reduction in both ceramide and S1P levels in Ishikawa and HEC-1A cells. However, the decrease in S1P was less pronounced in HEC-1A cells, suggesting a cell type-specific regulation of sphingolipid metabolism. SPT silencing led to increased cell viability in both cell lines, with a concomitant and significant reduction in cell death observed in Ishikawa cells. Additionally, a decrease in caspase-3 activity was observed in Ishikawa_(-SPT)_ cells. Importantly, SPT gene silencing also resulted in a significant reduction in the percentage of cells with mitochondrial depolarization in both cell lines, with a more pronounced decrease observed in Ishikawa cells. These findings further support the hypothesis that ceramides play a crucial role in promoting mitochondrial dysfunction and apoptosis, particularly in Ishikawa cells. In contrast, the effects of ceramide depletion were less pronounced in HEC-1A cells, which exhibit a low baseline level of cell death, likely due to their reduced reliance on sphingolipid-mediated regulation of this process.

Another important observation in our study was the increase in ROS levels in both cell types following SPT silencing. However, despite this increase, ROS levels remained within a low range that is more likely to support cell proliferation rather than induce oxidative damage [50]. Additionally, the total antioxidant capacity significantly decreased in Ishikawa cells, which initially exhibited higher antioxidant reserves, including elevated catalase activity. Notably, catalase activity also decreased following SPT silencing in Ishikawa cells, whereas no significant changes were observed in HEC-1A cells. These changes were accompanied by alterations in the sphingolipid rheostat. Although we did not observe significant changes in the total sphingolipid rheostat (S1P/total ceramides) following gene silencing, we detected distinct shifts in the rheostat values for specific ceramide species. Notably, an increase in the sphingolipid rheostat was observed in both cell types for C16:0-Cer and C18:0-Cer, which are known to play specialized roles in cellular signaling and survival [51]. Furthermore, Ishikawa cells exhibited elevated sphingolipid rheostat values for C18:1-Cer and C24:0-Cer, while HEC-1A cells showed increased values specifically for C24:1-Cer. These ceramide-specific differences in the S1P/ceramide ratio may contribute to the distinct regulation of survival and death pathways in each cell type, as individual ceramide species exert unique biological effects. Certain ceramides may promote pro-survival signaling or modulate cellular stress responses in a way that counteracts the pro-apoptotic effects of others [52]. Our correlation analyses further support this observation. In Ishikawa cells, we observed stronger associations between specific ceramide rheostat values and cell death than in HEC-1A cells. Notably, rheostat values involving S1P/C14:0-Cer, S1P/C16:0-Cer, S1P/C20:0-Cer, and S1P/C22:0-Cer showed moderate to strong positive correlations with cell death, whereas rheostats involving S1P/C18:1-Cer, S1P/C18:0-Cer, and S1P/C24:0-Cer exhibited negative correlations. These findings are consistent with the literature indicating that C18:0-Cer plays a pro-apoptotic role, while C16:0-Cer may support cell survival. Our data reinforce this distinction, suggesting that the biological impact of the sphingolipid rheostat on cell fate is strongly dependent on the specific ceramide species. Moreover, our results highlight a tighter coupling between ceramide metabolism and apoptotic regulation in type 1 EC (Ishikawa) cells compared to type 2 EC (HEC-1A) cells.

While this study provides valuable insights using well-established endometrial cancer cell models, certain limitations should be acknowledged. Although Ishikawa and HEC-1A cells are widely used to represent type 1 and type 2 EC, respectively, they may not fully reflect the molecular heterogeneity observed in clinical tumors. In particular, HEC-1A cells may not capture the transcriptomic complexity of high-grade serous carcinomas [53]. Additionally, the lack of a non-cancerous endometrial cell line limits the interpretation of the results. Future research should include the use of patient-derived tissues from both types of endometrial cancer, as well as healthy endometrial cells, to validate and extend these findings.

## 4. Materials and Methods

### 4.1. Cell Culture Experiments

The human endometrial cancer cell line Ishikawa (representing type 1 endometrial cancer) was purchased from Merck, while HEC-1-A (representing type 2 endometrial cancer) was obtained from the American Type Culture Collection (ATCC). Both cell lines were cultured in MEM (Gibco, New York, NY, USA) supplemented with 10% fetal bovine serum (FBS, Gibco), streptomycin (50 µg/mL), and penicillin (50 units/mL; Gibco). The media were further enriched with albumin-conjugated free fatty acids (150 µM), specifically oleic and palmitic acids, in a 2:1 molar ratio. Cells were maintained under standard humanified conditions (37 °C, 5% CO_2_), and experiments were conducted using cells from the third to sixth passages.

To silence the expression of the *Sptlc1* and *Sptlc2* genes, we utilized a small interfering RNA (siRNA)-mediated RNA interference approach. Briefly, 250 × 10^3^ cells were seeded in 6-well plates and transfected with 10 nM/L of MISSION^®^ esiRNA targeting human *Sptlc1* (EHU082241) and *Sptlc2* (EHU120701), along with 10 µL of transfection reagent per well (both from Sigma-Aldrich, Saint Louis, MO, USA). Cells with silenced *Sptlc1* and *Sptlc2* genes were designated as Ishikawa_(-SPT)_ and HEC-1-A_(-SPT)_, while cells transfected with human eGFP served as controls (Ishikawa_(CON)_ and HEC-1-A_(CON)_). After 72 h of transfection, cells and culture media were collected for subsequent analyses. All experiments were performed in nine independent replicates (*n* = 9).

### 4.2. Evaluation of Cell Death

Cell cultures were prepared as described above. Supernatants were collected for the detection of histone release in apoptotic bodies using a commercially available ELISA kit (Cell Death Detection ELISA Plus, Roche, Indianapolis, IN, USA). The assay was performed according to the manufacturer’s protocol, and absorbance was measured at 405 nm using a VarioscanLux instrument (Thermo Fisher, Waltham, MA, USA).

### 4.3. Evaluation of Cell Viability

Cell viability was determined by MTT [49] assay with the use of a commercially available kit (Cell Proliferation Kit I (MTT), Roche, Indianapolis, IN, USA). The assay was performed according to the manufacturer’s protocol. Results were acquired using the VarioscanLux instrument (Thermo Fisher, Waltham, MA, USA).

### 4.4. Real-Time PCR

Total RNA was isolated from cells using the mirVana Isolation Kit (Thermo Scientific, Waltham, MA, USA) following the manufacturer’s instructions. cDNA synthesis was performed using the Transcriptor First Strand cDNA Synthesis Kit (Roche, Mannheim, Germany). Real-time PCR was conducted using the following primers: SPTLC1: Forward, 5′-CGGAAGGAAGCGGCTAACTA-3′; Reverse, 5′-GGTAAGCAGGAGCCTCGTAA-3′. SPTLC2: Forward, 5′-GGGAAGTACGGAACGGGTAC-3′; Reverse, 5′-TTTTGTATTTGACAGCCAGC-3′. PPIA-peptidylprolyl isomerase A (housekeeping gene): Forward, 5′-AGCTGTTTACCCCTGATCGTG-3′; Reverse, 5′-CCTTGTCTGCAAACAGAAGGC-3′. Amplification was performed using a LightCycler480 system (Roche, Mannheim, Germany). Relative gene expression levels were calculated using the 2^ΔΔCt^ method and normalized to PPIA expression.

### 4.5. Western Blotting

Proteins were extracted from cells using RIPA buffer (Sigma-Aldrich, Saint Louis, MO, USA) containing 0.5 mM TCEP (Sigma-Aldrich, Saint Louis, MO, USA) and protease/phosphatase inhibitors (cOmplete ULTRA mini and PhosSTOP tablets, Roche). Protein concentrations were determined using the Pierce 660 nm Protein Assay Kit (Thermo Fisher Scientific, Waltham, MA, USA), with fatty acid-free bovine serum albumin as the standard. Proteins (30 µg) were denatured in Laemmli buffer, separated by SDS-PAGE (Criterion Cell electrophoresis system and Criterion TGX midi Any kD gel), and transferred to PVDF membranes using a BioRad Trans Blot SD semi-dry transfer system. Membranes were incubated with primary antibodies targeting SPT1, SPT2, and GAPDH (Cell Signaling Technology, Danvers, MA, USA). After incubation with HRP-conjugated secondary antibodies, protein bands were visualized using Clarity™ Western ECL chemiluminescent substrate (Bio-Rad) and imaged with a Bio-Rad ChemiDoc XRS+ system. Band intensities were quantified using Bio-Rad Image Lab 6.0 software and normalized to GAPDH expression. Results were expressed as fold changes relative to the control group. Unless otherwise specified, all reagents and equipment for immunoblotting were obtained from Bio-Rad (Hercules, CA, USA).

### 4.6. Sphingolipid Measurements

Sphingolipid levels were quantified using ultra-high-performance liquid chromatography coupled with tandem mass spectrometry (UHPLC/MS/MS), as described by Blachnio-Zabielska et al. [54], with minor modifications. Cells were homogenized in a buffer containing 0.25 M sucrose, 25 mM KCl, 50 mM Tris, and 0.5 mM EDTA (pH 7.4). Protein concentrations were determined using the Pierce660nm Protein Assay Kit (Thermo Fisher Scientific, Waltham, MA, USA). Internal standards (Sph-d7, SPA-d7, S1P-d7, and deuterated ceramides; Avanti Polar Lipids, Alabaster, AL, USA) were added to each sample, followed by extraction with a mixture of isopropanol, water, and ethyl acetate (30:10:60, *v*:*v*:*v*). After sonication and centrifugation (4000 rpm, 10 min, 4 °C), samples were dried under nitrogen and reconstituted in LC Solvent B (2 mM ammonium formate, 0.1% formic acid in methanol). Chromatographic separation was performed on a Zorbax SB-C8 column (2.1 × 150 mm, 1.8 μm; Agilent Technologies, Santa Clara, CA, USA) using a binary gradient. Sphingolipids were detected using a Thermo TSQ Altis Plus mass spectrometer (Thermo Fisher, Waltham, MA, USA) in selected reaction monitoring (SRM) mode, with quantification based on standard curves for each compound.

### 4.7. Caspase-3 Assay

Caspase-3 activity was measured using the Caspase-3 Assay Kit, Fluorometric (MAK457, Sigma-Aldrich) according to the manufacturer’s instructions. Cells were seeded in sterile black flat-bottom 96-well plates and incubated overnight. After removing the culture medium, 100 µL of the working reagent containing assay buffer, DTT, and the DEVD-AFC substrate was added to each well. Plates were incubated for 60 min at 37 °C in the dark. Fluorescence was measured at 400 nm excitation and 490 nm emission using a microplate reader (Varioskan Lux, Thermo Scientific, Waltham, MA, USA). Caspase-3 activity was expressed as relative fluorescence units (RFUs) after subtracting background signals from control wells.

### 4.8. Mitochondrial Membrane Potential Assay

Mitochondrial membrane potential was assessed using the Muse^®^ MitoPotential Kit (Luminex Corporation, Austin, TX, USA) following the manufacturer’s instructions. The assay specifically identifies cells exhibiting mitochondrial depolarization. The total percentage of cells with depolarized mitochondria was calculated based on the combined populations displaying loss of mitochondrial membrane potential.

### 4.9. Catalase Activity Assay

Catalase activity was measured using the Invitrogen™ Catalase Colorimetric Activity Kit (Catalog number EIACATC, Thermo Fisher Scientific) according to the manufacturer’s instructions. Cells were homogenized using the kit-provided buffer, and then centrifuged at 10,000× *g* for 15 min at 4 °C. The supernatants were collected and used in further procedures. Absorbance was measured at 560 nm using a microplate reader (Varioskan Lux, Thermo Scientific), and catalase activity was expressed as units per mL.

### 4.10. ROS Concentration

Reactive oxygen species (ROS) levels in Ishikawa and HEC-1A cells were quantified using a commercially available ELISA kit (EK710415, AFG Bioscience, Northbrook, IL, USA) according to the manufacturer’s protocol. Cell lysates were prepared by resuspending the cells in lysis buffer, followed by centrifugation at 1500× *g* for 10 min at 4 °C. The resulting supernatant was collected for subsequent analysis. A micro-ELISA strip plate pre-coated with an anti-ROS antibody was utilized for the assay. Standards and samples were added to the designated wells, followed by incubation with a horseradish peroxidase (HRP)-conjugated anti-ROS antibody. After thorough washing, 3,3′,5,5′-tetramethylbenzidine (TMB) substrate was added to initiate the enzymatic reaction. The reaction was terminated, resulting in a colorimetric change. The optical density (OD) was measured at 450 nm using a microplate reader, and the ROS concentration was determined by extrapolating the values from a standard curve.

### 4.11. Antioxidant Capacity (TAC)

The total antioxidant capacity (TAC) of the samples was determined colorimetrically based on the ability to neutralize the ABTS⁺ radical cation [2,2-azino-bis-(3-ethylbenzothiazoline-6-sulfonate)]. Absorbance changes were measured at 660 nm, and TAC levels were calculated using a Trolox standard curve [55].

### 4.12. Statistical Analyses

All statistical analyses were performed using GraphPad Prism 9 (GraphPad Software, La Jolla, CA, USA). Data are presented as medians with interquartile ranges from four independent experiments (*n* = 9). Significant differences were identified using the nonparametric Mann–Whitney test, with a significance threshold of *p* < 0.05. The relationships between variables were examined by means of Pearson correlation.

## 5. Conclusions

Our findings reveal distinct differences in sphingolipid metabolism between Ishikawa (type 1) and HEC-1A (type 2) endometrial cancer cells, which correspond to their divergent phenotypic behaviors. Ishikawa cells, characterized by higher levels of pro-apoptotic ceramides and lower levels of pro-proliferative S1P, exhibited greater susceptibility to cell death and higher total antioxidant capacity. In contrast, HEC-1A cells, with lower ceramide levels and higher S1P levels, showed enhanced cell viability, consistent with their more aggressive phenotype. Silencing the genes encoding the subunits of serine palmitoyltransferase, the rate-limiting enzyme in the de novo sphingolipid synthesis pathway, led to a reduction in both ceramides and S1P levels in both cell types. This effect was more pronounced in Ishikawa cells, where it was associated with a marked decrease in cell death and total antioxidant capacity, suggesting a greater dependence on sphingolipid-mediated regulation of redox balance and apoptosis. These results underscore the important role of sphingolipid metabolism in modulating apoptosis, redox homeostasis, and cell survival across different endometrial cancer subtypes. Although our results provide important mechanistic insights, the therapeutic implications of targeting sphingolipid metabolism remain speculative. Future studies incorporating in vivo models and pharmacological interventions are essential to determine the feasibility, efficacy, and safety of manipulating this pathway as a potential therapeutic strategy for endometrial cancer.

## Figures and Tables

**Table 1:** The concentration of sphingolipids.

	ISHIKAWA_(CON)_	ISHIKAWA_(–SPT)_	HEC-1A_(CON)_	HEC-1A_(–SPT_)_
	Me (Q1–Q3) [pmol/mg]	Me (Q1–Q3) [pmol/mg]	Me (Q1–Q3) [pmol/mg]	Me (Q1–Q3) [pmol/mg]
Sph	18.0 (14.04–22.12)	4.4 (3.8–4.8) ****	8.5 (7.8–9.2) ^d^	2.5 (2.0–2.9) ****
SPA	46.4 (34.7–53.0)	25.3 (21.9–29.4) ****	27.2 (26.1–32.8) ^d^	19.2(17.4–20.5) ****
S1P	14.8 (13.4–16.5)	9.3 (8.5–10.2) ****	25.7 (23.5–27.2) ^d^	20.5(19.8–21.6) ***
C14:0-Cer	14.1 (13.3–14.6)	13.8 (12.6–15.1)	13.5 (11.9–13.9)	12.1(9.7–13.8)
C16:0-Cer	310.6 (286.6–327.7)	245.8 (238.7–269.4) ****	423.2 (361.9–442.4) ^c^	275.2 (253.8–288.3) ****
C18:1-Cer	9.5 (9.2–10.6)	5.3 (5.1–6.1) ****	5.2 (5.0–6.3) ^d^	4.4 (3.9–4.7) ***
C18:0-Cer	53.1 (51.4–54.6)	26.5 (24.5–31.0) ****	30.1 (29.3–32.2) ^d^	19.3 (17.8–20.5) ****
C20:0-Cer	21.1 (19.1–22.9)	21.5 (21.0–25.5)	20.3 (18.0–21.0)	22.5 (19.4–23.5) *
C22:0-Cer	18.0 (13.8–21.9)	16.5 (14.9–19.2)	20.5 (17.0–22.1)	28.7 (24.1–32.5) **
C24:1-Cer	314.5 (305.8–327.1)	184.4 (175.0–197.5) ****	99.8 (92.2–107.3) ^d^	51.11 (49.1–53.9) ****
C24:0-Cer	97.4 (90.3–107.1)	36.9 (34.1–39.8) ***	26.8 (25.3–28.4) ^d^	21.1 (18.4–22.7) ****
Total-Cer	845.6 (807.2–861.6)	551.3 (548.2–580.3) ****	641.8 (585.2–654.1) ^d^	426.2 (413.0–443.9) ****

Data are presented as medians (interquartile range). Analysis was performed with the Mann–Whitney U test: * *p* < 0.05, ** *p* < 0.01, *** *p* < 0.001, **** *p* < 0.0001 vs. control group; ^c^ *p* < 0.001, ^d^ *p* < 0.0001 vs. Ishikawa_(CON)_ group. Sph—Sphingosine, SPA—Sphinganine, S1P—Sphingosine-1-phosphate, C14:0-Cer—Ceramide with a 14 carbon acyl chain length, C16:0-Cer—Ceramide with a 16 carbon acyl chain length, C18:1-Cer—Ceramide with an 18 carbon acyl chain length with one carbon-carbon double bone, C18:0-Cer—Ceramide with an 18 carbon chain length, C20:0-Cer—Ceramide with a 20 carbon acyl chain length, C22:0-Cer—Ceramide with a 22 carbon acyl chain length, C24:1-Cer—Ceramide with a 24 carbon acyl chain length with one carbon-carbon double bone, C24:0-Cer—Ceramide with a 24 carbon acyl chain length, Total-Cer—Total ceramide concentration in the sample.

**Table 2:** Shingolipid rheostat.

	ISHIKAWA_(CON)_	ISHIKAWA_(–SPT)_	HEC-1A_(CON)_	HEC-1A_(–SPT_)_
	Me (Q1–Q3) [pmol/mg]	Me (Q1–Q3) [pmol/mg]	Me (Q1–Q3) [pmol/mg]	Me (Q1–Q3) [pmol/mg]
C14:0-Cer	1.06 (0.97–1.19)	0.64 (0.61–0.72) ****	1.94 (1.79–2.13) ^d^	1.76 (1.49–2.14)
C16:0-Cer	0.048 (0.045–0.053)	0.062 (0.055–0.073) **	0.037 (0.033–0.043) ^c^	0.078 (0.072–0.082) **
C18:1-Cer	1.559 (1.412–1.686)	1.721 (1.621–1.818) *	4.499 (4.188–4.985) ^d^	4.599 (4.459–5.302)
C18:0-Cer	0.272 (0.254–0.310)	0.345 (0.281–0.411) *	0.816 (0.765–0.890) ^d^	1.117 (0.964–1.202) ***
C20:0-Cer	0.675 (0.637–0.824)	0.408 (0.371–0.455) ****	1.280 (1.190–1.419) ^d^	0.909 (0.850–1.093) ****
C22:0-Cer	0.820 (0.744–0.977)	0.562 (0.531–0.584) ****	1.246 (1.189–1.299) ^d^	0.740 (0.668–0.819) ****
C24:1-Cer	0.047 (0.042–0.052)	0.053 (0.044–0.056)	0.254 (0.225–0.297) ^d^	0.392 (0.384–0.438) ****
C24:0-Cer	0.149 (0.140–0.172)	0.249 (0.232–0.269) ****	0.958 (0.880–1.030) ^d^	0.962 (0.902–1.151)
Total-Cer	0.018 (0.016–0.019)	0.017 (0.015–0.019)	0.043 (0.036–0.044) ^d^	0.049 (0.045–0.050)

Data are presented as medians (interquartile range). Analysis was performed with the Mann–Whitney U test: * *p* < 0,05, ** *p* < 0.01, *** *p* < 0.001, **** *p* < 0.0001 vs. control group; ^c^ *p* < 0.001, ^d^ *p* < 0.0001 vs. Ishikawa_(CON)_ group. C14:0-Cer—Ceramide with a 14 carbon acyl chain length, C16:0-Cer—Ceramide with a 16 carbon acyl chain length, C18:1-Cer—Ceramide with an 18 carbon acyl chain length with one carbon-carbon double bone, C18:0-Cer—Ceramide with an 18 carbon chain length, C20:0-Cer—Ceramide with a 20 carbon acyl chain length, C22:0-Cer—Ceramide with a 22 carbon acyl chain length, C24:1-Cer—Ceramide with a 24 carbon acyl chain length with one carbon-carbon double bone, C24:0-Cer—Ceramide with a 24 carbon acyl chain length, Total-Cer—Total ceramide concentration in the sample.

**Table 3:** Correlation between the shingolipid rheostat and cell death.

	ISHIKAWA	HEC-1A
S1P/C14:0-Cer	r = 0.61 *p* = 0.0076 **	r = −0.0002 *p* = 0.9995
S1P/C16:0-Cer	r = 0.47 *p* = 0.0491 *	r = −0.07 *p* = 0.7943
S1P/C18:1-Cer	r = −0.71 *p* = 0.0011 **	r = −0.004 *p* = 0.9861
S1P/C18:0-Cer	r = −0.61 *p* = 0.0079 **	r= −0.45 *p* = 0.0623
S1P/C20:0-Cer	r = 0.50 *p* = 0.0357 *	r = 0.38 *p* = 0.1190
S1P/C22:0-Cer	r = 0.57 *p* = 0.0133 *	r = 0.50 *p* = 0.0350 *
S1P/C24:1-Cer	r = −0.40 *p* = 0.0997	r = −0.43 *p* = 0.0745
S1P/C24:0-Cer	r = −0.86 *p* = <0.0001 ****	r = −0.29 *p* = 0.2466
S1P/Tota-Cer	r = 0.002 *p* = 0.9930	r = −0.15 *p* = 0.4725

Data are presented as Pearson correlation coefficient. Statistical significance: * *p* < 0.05, ** *p* < 0.01, **** *p* < 0.0001.

**Table 4:** Mitochondrial membrane potential changes after SPT Gene-Silencing in EC Cells (Muse Mitopotential Assay).

	ISHIKAWA _(CON)_	ISHIKAWA _(–SPT)_	*p*-Value	HEC-1A _(CON)_	HEC-1A _(–SPT_)_	*p*-Value
	Me (Q1–Q3) [pmol/mg]	Me (Q1–Q3) [pmol/mg]		Me (Q1–Q3) [pmol/mg]	Me (Q1–Q3) [pmol/mg]	
Depolarized/ Live (LL)	17.50 (16.95–19.65)	15.55 (15.10–17.95) *	0.049	19.00 (17.30–21.95)	17.65 (14,20–19.10)	0.087
Depolarized/ Dead (UL)	26.20 (25.10–28.52)	15.35 (14.75–16.30) ****	<0.0001	14.50 (13.85–15.80)	12.47 (11.55–13.15) *	0.021
Total Depolarized	45.80 (42.05–46.10)	31.40 (30.50–33.25) ****	<0.0001	34.80 (31.60–36.80)	28.46 (27.35–30.65) **	0.003

Data are presented as medians (interquartile range). Analysis was performed with the Mann–Whitney U test: * *p* < 0.05, ** *p* < 0.01, **** *p* < 0.0001 indicate significant differences from the controls.

## Data Availability

The article contains complete data used to support the findings of this study.

## Abbreviations

The following abbreviations are used in this manuscript:

EC	Endometrial cancer
ELISA	Enzyme-Linked Immunosorbent Assay
GAPDH	Glyceraldehyde-3-phosphate dehydrogenase
RIPA buffer	Radioimmunoprecipitation buffer
ROS	Reactive oxygen species
S1P	Sphingosine-1-phosphate
SPT	Serine palmitoyltransferase
*Sptlc1*	Gene encoding serine palmitoyltransferase, long chain base subunit-1
*Sptlc2*	Gene encoding serine palmitoyltransferase, long chain base subunit-2
TAC	Total antioxidant capacity
UHPLC/MS/MS	Ultra-high-performance liquid chromatography tandem mass spectrometry
